# Burden and factors associated with clinical neonatal sepsis in urban Uganda: a community cohort study

**DOI:** 10.1186/s12887-018-1323-4

**Published:** 2018-11-13

**Authors:** Violet Okaba Kayom, Jamiir Mugalu, Abel Kakuru, Sarah Kiguli, Charles Karamagi

**Affiliations:** 1 Department of Paediatrics and Child Health, Makerere University College of Health Sciences, P.O. Box 7062, Kampala, Uganda; 2 Mulago National Referral Hospital, P.O. Box 7051, Kampala, Uganda; 3 Infectious Diseases Research Collaboration, P.O.Box 7475, Kampala, Uganda

**Keywords:** Neonate, Sepsis, Community

## Abstract

**Background:**

Neonatal sepsis is one of the most important causes of mortality in developing countries and yet the most preventable. In developing countries clinical algorithms are used to diagnose clinical neonatal sepsis because of inadequate microbiological services. Most information on incidence and risk factors of neonatal sepsis are from hospital studies which may not be generalized to communities where a significant proportion of mothers do not deliver from health facilities. This study, conducted in urban Uganda, sought to determine the community based incidence of clinical neonatal sepsis and the factors associated.

**Methods:**

This was a cohort of mother-neonate pairs in Kampala, Uganda from March to May 2012. The enrolled neonates were assessed for clinical sepsis and factors associated, and followed up till the end of the neonatal period. STATA version 10 was used to analyse the data.

**Results:**

The community based incidence of neonatal sepsis was 11% (95% CI: 7.6–14.4). On bivariate analysis, lack of financial support from the father (OR 4.09, 95% CI 1.60–10.39) and prolonged rupture of membranes more than 18 h prior to delivery (OR 11.7, 95% CI 4.0–31.83) were significantly associated with neonatal sepsis. Maternal hand washing prior to handling the baby was found to be protective of neonatal sepsis (OR 0.41, 95% CI 0.18–0.94). Of the 317 infants who completed the follow up period, one died within the neonatal period giving a neonatal mortality of 0.003%.

**Conclusion:**

The high incidence of clinical neonatal sepsis in this urban community with high rates of antenatal care attendance and health facility delivery places a demand on the need to improve the quality of antenatal, perinatal and postnatal care in health facilities with regards to infection prevention including promoting simple practices like hand washing. The astoundingly low mortality rate is most likely because this was a low risk cohort. However it may also suggest that the neonatal mortality in developing countries may be reduced with promotion of simple low cost interventions like community follow up of neonates using village health teams or domiciliary care.

## Background

Every year about 2.9 million neonates die worldwide and most of these deaths occur in low resource settings [1]. In Sub-Saharan Africa (SSA) the neonatal mortality rate (NMR) is estimated to be 29 per 1000 live births with Uganda grappling with a high rate of 27 per 1000 live births [1, 2]. Majority of these neonates are dying from intra-partum related complications, prematurity and its complications and sepsis. Most of the morbidity and mortality from sepsis are preventable and therefore knowledge on the burden of the problem is very important.

Currently there is no universal agreement on the definition of neonatal sepsis due to the variability in diagnostic criteria. Neonatal sepsis has been commonly described as a clinical syndrome characterised by signs and symptoms of infection with or without accompanying bacteraemia in the first month of life. It encompasses septicaemia, meningitis, pneumonia, arthritis, osteomyelitis and urinary tract infection [3]. The presence of a positive blood culture is the gold standard for diagnosis of neonatal sepsis. Although this is the case most studies have shown that in neonates with clinical and laboratory features consistent with neonatal sepsis, the majority have negative blood culture results. In developing countries the challenge of diagnosis is further worsened by lack of reliable microbiological investigations, therefore necessitating the use of clinical criteria in identifying neonates with possible sepsis.

The World Health Organisation (WHO) young infant study group developed simple clinical criteria to identify neonates with signs of severe bacterial infection who need referral to the health facility [4]. These criteria have been adapted in the Integrated Management of Neonatal and Childhood Illness (IMNCI) clinical algorithm which uses the following clinical features to make a diagnosis of clinical neonatal sepsis: if the neonate had temperature more than 37.5’C or felt hot to touch, convulsions (by history), fast breathing (> 60 breaths/minute), severe chest in drawing, nasal flaring, grunting, bulging fontanelle, pus draining from ear, umbilical redness extending to the skin, feels cold (by history), many or severe skin pustules, difficult to wake up, cannot be calmed within 1 h, less than normal movement, not able to feed and not able to attach to breast or suck [5].

Although this algorithm has a high sensitivity and low specificity it is able to identify a significant percentage of neonates with sepsis and has been widely used for clinical and research purposes in low resource settings.

The incidence of neonatal sepsis reported from studies varies widely due to differences in population studied, diagnosis criteria and the case definition. It is estimated that in 2012 about 6.9 million neonates were diagnosed with possible serious bacterial infection needing treatment and 2.6 million of these occurred in SSA [6]. A review of 32 studies reported that neonatal infections may be responsible for 8–80% of neonatal deaths and clinically diagnosed neonatal sepsis incidence of up to 170 per 1000 live births [7]. Most of the incidences reported are derived from hospital studies which may not reflect the true picture in a setting like Uganda where a significant proportion of mothers do not deliver from health facilities (43%) or seek treatment for their sick infants from health facilities [8]. For those who deliver from health facilities more often than not there is early postnatal discharge. Thus neonates born at home or treated from home may not be accounted for. Community based incidence would therefore provide a better representation of the burden of the disease.

Several factors have been found to put a neonate at risk of acquiring sepsis. These factors span from in-utero, peri-partum and postpartum factors, including newborn factors and the home and community where the baby is raised. [9–11]. The magnitude of the factors may vary from place to place and so it’s important to know the community specific factors.

Estimation of the burden of neonatal sepsis is important in planning for the health care system’s response to the sick neonates in terms of personnel, commodities and in-patient care. Data on factors associated with neonatal sepsis would help in identifying high risk neonates and thus prioritising the limited resources to where they are most needed. Such information from a community perspective is vital for setting up community interventions for prevention of neonatal sepsis and thus guiding policy towards achieving the post 2015 agenda of ending preventable child deaths [12].

This study therefore sought to determine the burden and the factors associated with clinical neonatal sepsis in an urban community in Uganda.

## Methods

### Study area

The study was conducted in Kawempe division, an urban community in the northwestern part of Kampala, the capital city of Uganda. Kawempe division has an area of 32.45 km^2^ and population estimates of 268,659 of which 52% are female. It is densely populated and has areas characterised by uncontrolled developments and slum conditions [13]. It is served by 3 government health facilities, one private-not-for-profit hospital and several privately owned clinics which provide curative services.

### Sample size calculation

The sample size was calculated using the modified Kish Leslie formula for sample size estimation;

$$ \mathrm{N}=\frac{{\mathrm{Z}}^{2\ast}\mathrm{P}\ \left(1\hbox{-} \mathrm{P}\right)\ast \mathrm{Deff}}{{\mathrm{D}}^2} $$

Where:

- N - Sample size required
- Z - Standard normal value corresponding to 95% confidence interval (1.96)
- D - Absolute error between the estimated and true value = 0.05 (5%)
- P - Estimated incidence of neonatal sepsis in Kawempe division. An incidence of 17% was used as found in the community based prospective observational study in India by Bang et al. 2001 [14].

Deff: Design effect taken to be 1.5

Hence, *N* = 325 neonates

### Number of clusters

The number of clusters, C, that was studied was calculated from the formula by Bennett S et al. 1991 [15]

$$ \mathrm{C}=\mathrm{P}\ \left(1-\mathrm{P}\right)\mathrm{D}/{\mathrm{S}}^2\mathrm{b} $$

Where:

- P: incidence (17%)
- D: Design effect (1.5)
- S: Standard error given by confidence interval/Z alpha (0.05/1.96 = 0.0255)
- b: number of responses per cluster set at 10 for convenience

The estimated number of clusters was 33.

To allow for non-response a total of 34 clusters was studied. Thus the sample size calculated was **335** households with neonates.

### Study design, participants and duration

This was a population based cohort study with both retrospective and prospective components. The retrospective component consisted of the history of the condition of the neonate from birth to the point of contact with the research team, while the prospective component included the follow up period till the end of the neonatal period.

The study participants included mother-neonate pairs living within Kawempe division during the study period who consented to participating in the study. The study enrolled neonates from birth to 28 days of age. Neonates with gross congenital malformation and extremely low birth weight were excluded from the study because their presentation may simulate symptoms of clinical neonatal sepsis.

### The study was conducted from February to June 2012

#### Study procedures

Thirty four out of 119 zones within Kawempe division in Kampala district were sampled using probability proportional to size. The principal investigator and two research assistants (study team) contacted the Local Council 1 chairpersons and village health teams (VHTs) of the zones and held meetings to explain the research. The study team moved with the VHTs in the zones, and in each zone a total of 10 households with neonates were consecutively enrolled in the study. Informed consent was obtained from eligible mothers. A neonate aged 0 to 28 days of age who met the selection criteria was enrolled in the study. A pretested questionnaire was used to obtain history, physical examination and evaluate factors associated with neonatal sepsis. These included maternal factors, delivery and newborn care practices, and household factors. The newborn care practices assessed included cord care, skin care, washing of the hands prior to handling the baby, early initiation and exclusive breastfeeding and thermal protection.

The WHO IMNCI criteria were applied to assess babies for clinical sepsis [5]. The IMNCI criteria uses the following clinical features to make a diagnosis of clinical neonatal sepsis: if the neonate had temperature more than 37.5’C or felt hot to touch, convulsions (by history), fast breathing (> 60 breaths/minute), severe chest in drawing, nasal flaring, grunting, bulging fontanelle, pus draining from ear, umbilical redness extending to the skin, feels cold (by history), many or severe skin pustules, difficult to wake up, cannot be calmed within 1 h, less than normal movement, not able to feed and not able to attach to breast or suck.

A retrospective review of the history was taken to find out if the neonate had the symptoms suggestive of neonatal sepsis since birth. A conclusion of clinical neonatal sepsis was ascertained if the baby had two or more symptoms of sepsis listed in the IMNCI criteria and had been reviewed or admitted in a health unit. Medical documents from the health units attended were also used to get information on presentation of the patient to the health units and the treatment received.

Neonates diagnosed with clinical neonatal sepsis were referred to the emergency unit of the national referral hospital (Mulago hospital). All the mothers enrolled were availed the telephone contacts of the principal investigator and research assistants and informed to call the research team in case of symptoms of neonatal illness. Most of the mothers whose neonates had symptoms suggestive of sepsis took their babies to the national referral hospital. However a few opted for care in private clinics.

The study outcome was ascertained after 28 days of life. The study team made another visit to the homes of enrolled infants and inquired if the infants had developed symptoms suggestive of sepsis which were not reported to the study team since the last contact with the research team. Mothers who did not contact the study team when their babies were ill were asked about the symptoms the baby had. The medical records of the babies, where available, were also reviewed. The study team made telephone calls to mothers who had changed location or those not found at home at the end of the follow up period.

#### Data management and analysis

Questionnaires were checked daily for completeness and correctness. All data was double entered, cleaned, edited, coded and double entered into ACCESS data base 2007 and exported to STATA version 10 for analysis. Univariate analysis was used to get the general description of the data. Categorical variables were summarised into percentages and proportions. The continuous variables were summarised into means, medians, standard deviation and ranges for description. The incidence of clinical neonatal sepsis was obtained by calculating the proportion of neonates with symptoms and signs of clinical neonatal sepsis out of the total number of neonates who completed the study. Bivariate analysis was used to determine association between neonatal sepsis and various independent variables including maternal factors, perinatal factors and the newborn care practices. Continuous independent variables were categorised and associations established using Chi-squared tests. This was similarly done for categorical variables. Odds ratio was used as a measure of strength of association for categorical variables. *P*-value of less than 0.05 and 95% confidence limit not including one were used as tests for statistical significance.

Multivariate analysis was done to assess for interaction and confounding of the independent variables with respect to the main predictor. Factors with P-value of 0.2 or less at bivariate analysis were selected for further multivariate analysis.

#### Study profile

During the study period a total of 353 neonates were screened and of these 15 were excluded from the study (8 did not consent to participate in the study and 7 planned to move out of the study area before the end of the neonatal period). Of the 338 subjects enrolled, 317 completed the follow up period. Twenty one (6%) of the neonates enrolled were lost to follow up. The demographic characteristics of the neonates who were lost to follow up were not significantly different from those who completed the study. The main reason for the loss to follow up was change in residential location and the absence of a functioning telephone contact.

## Results

### Description of the study participants

Three hundred and thirty eight (338) mother-neonate pairs in Kawempe division were enrolled in the study from March to May 2012.

Majority of the neonates were born at term with mean gestational age of 39.7 weeks (sd 2.3) and mean birth weight of 3.4 kg (sd 0.7). The neonates were almost equally distributed by gender with males comprising 156 (46.2%). The mean neonatal age at enrolment was 14.8 days (sd 8.5).

The mean maternal age of the study participants was 25.4 years (sd 5.4). Twenty six percent of the maternal study participants were prime gravida and another 26% had parity of 4 and above. Most of the maternal participants were married (85%) and received financial support from their husbands (91%). Fifty nine percent of the mothers had attained secondary or tertiary level of education, 36% only primary level of education while 4% had no formal education. Other maternal, antenatal and perinatal characteristics are shown in Table 1.

**Table 1:** Maternal, antenatal and perinatal characteristics of subjects in Kampala District, 2012

Variable	Frequency (n)	Percentage (%)
Attended ANC		
Yes	336	99.4
No	2	0.6
Number of ANC attendance		
< 4 times	160	51
≥ 4 times	154	49
HIV Status		
Negative	311	92.8
Positive	13	3.9
Unknown	11	3.3
Had Fever during pregnancy		
No	165	48.8
Yes	173	51.2
Abnormal PV discharge during pregnancy		
No	225	66.6
Yes	113	33.4
Pain when passing urine		
No	249	73.7
Yes	89	26.3
Bleeding during pregnancy		
No	316	93.8
Yes	21	6.2
Place of delivery		
Hospital	209	61.8
Health centre	70	20.7
Clinic/Maternity	38	11.3
Home	21	6.2
Duration of rupture of membranes		
Less than 18 h	292	93
More than 18 h	22	7

### Incidence of neonatal sepsis and the mortality rate

Of the 317 subjects who were followed up till the end of the neonatal period, a total of 35 developed clinical neonatal sepsis, giving an incidence of 11%. Of the neonates who developed sepsis 16 (45.7%) were males. More (57%) neonates developed sepsis within the first 7 days of life. Of the 317 infants who completed the follow up period, one died within the neonatal period giving a neonatal mortality of 0.003%.

### Factors associated with neonatal sepsis

Under bivariate analysis lack of financial support from the father was found to be significantly associated with neonatal sepsis (OR 4.09, 95% CI 1.60–10.39). The neonates of mothers who had prolonged rupture of membranes (PROM) more than 18 h prior to delivery were more likely to develop sepsis (OR 11.7, 95% CI 4.0–31.83). Hand washing with soap prior to handling the baby was found to be protective of neonatal sepsis (OR 0.41, 95% CI 0.18–0.94). Results for association with other parameters are indicated in Tables 2 and 3.

**Table 2:** Unadjusted association between neonatal sepsis and maternal factors among mother-infant dyads in Kampala District, 2012

Variable	Neonatal sepsis		OR(95% CI)	*P*- value
	Yes n (%)^a^	No n (%)^a^		
Maternal age in years				
15–25 years	21 (60.0)	153	1	
26–41 years	14 (40.0)	129	0.79 (0.39–1.62)	0.520
Maternal education				
None or Primary	16 (45.7)	110 (39.0)	1	
Secondary	15 (42.9)	138 (48.9)	0.75 (0.35–1.58)	0.444
Tertiary	4 (11.4)	34 (12.1)	0.81 (0.25–2.59)	0.721
Paternal financial support				
Yes	27 (77.1)	262 (93.2)	1	
No	8 (22.9)	19 (06.8)	4.09 (1.60–10.39)	0.0013^∞^
Attended antenatal				
< 4 times	17 (50.0)	143 (51.1)	1	
≥ 4 times	17 (50.0)	137 (48.9)	1.04 (0.51–2.13)	0.906
Fever during pregnancy				
No	18 (51.4)	135 (48.8)	1	
Yes	17 (48.6)	147 (51.2)	0.87 (0.43–1.75)	0.692
Abnormal PV discharge				
No	26 (74.3)	184 (65.3)	1	
Yes	9 (25.7)	98 (34.7)	0.65 (0.29–1.45)	0.287
Dysuria during pregnancy				
No	28 (80.0)	206 (73.0)	1	
Yes	7 (20.0)	76 (27.0)	0.68 (0.28–1.62)	0.378
PV Bleeding during pregnancy				
No	32 (91.4)	263 (93.6)	1	
Yes	3 (08.6)	18 (06.4)	1.37 (0.38–4.92)	0.628
Place of delivery				
Health facility	32 (91.4)	209 (95.4)	1	
Not health facility	3 (08.6)	13 (04.6)	1.94 (0.52–7.20)	0.313
Duration of rupture of membranes				
< 18 h	23 (67.6)	269 (96.1)	1	
≥ 18 h	11 (32.4)	11 (03.9)	11.7 (4.30–31.83)	< 0.0001^∞^

*OR* Odds ratio, *PV* Par vaginal

∞Significant *p* value

^a^Column percentage

**Table 3:** Unadjusted association between neonatal sepsis and newborn care practices among mother-infant dyads in Kampala District, 2012

Variable	Neonatal sepsis		OR (95% CI)	*P*- value
	Yes n (%)^a^	No n (%)^a^		
Applied substance on the cord				
No	28 (80.0)	223 (79.1)	1	
Yes	7 (20.0)	59 (20.9)	0.94 (0.39–2.27)	0.899
No of times the cord is cleaned per day				
< 3 times	14 (40.0)	115 (40.9)	1	
≥ 3 times	21 (60.0)	166 (59.1)	1.04 (0.51–2.13)	0.917
Bathed baby within 24 h				
No	20 (58.2)	164 (58.8)	1	
Yes	14 (41.2)	115 (41.2)	1.00 (0.48–2.06)	0.996
Washed hands prior to handling the baby				
No	10 (29.4)	41 (14.7)	1	
Yes	24 (70.6)	238 (85.3)	0.41 (0.18–0.94)	0.029^∞^
Bathed baby with herbal medicine				
No	9 (26.5)	97 (35.7)	1	
Yes	25 (73.5)	175 (64.3)	1.54 (0.69–3.43)	0.291

*OR* Odds ratio

^∞^Significant *p* value

^a^Column percentages

Variables considered for multivariate analysis included duration of rupture of membranes prior to delivery, paternal support and maternal hand washing prior to handling the baby. Maternal education was also included because it is a known confounder. Under multivariate analysis factors found to be independently associated with neonatal sepsis included lack of financial support from the father (OR 3.87, CI 1.40–10.68) and prolonged rupture of membranes more than 18 h prior to delivery (OR 12.6, CI 4.74–33.64) (Table 4).

**Table 4:** Adjusted association between neonatal sepsis and independent factors among mother-infant dyads in Kampala District, 2012

Characteristics	Neonatal sepsis		Adjusted Odds ratio (95% CI)^a^	*P*-Value
	Yes n (%)	No n (%)		
Maternal education				
None or Primary	16 (45.7)	110 (39.0)	1	
Secondary	15 (42.9)	138 (48.9)	0.82 (0.36–1.88)	0.637
Tertiary	4 (11.4)	34 (12.1)	0.67 (0.15–2.90)	0.591
Paternal financial support				
Yes	27 (77.1)	262 (93.2)	1	
No	8 (22.9)	19 (06.8)	3.87 (1.40–10.68)	0.009^∞^
Duration of rupture of membranes				
< 18 h	23 (67.6)	269 (96.1)	1	
≥ 18 h	11 (32.4)	11 (03.9)	12.6 (4.74–33.64)	< 0.001^∞^
Wash hands prior to handling the baby				
No	10 (29.4)	41 (14.7)	1	
Yes	24 (70.6)	238 (85.3)	0.50 (0.20–1.24)	0.135

^∞^Significant *p* value

^a^adjusted for maternal education, family support, duration of rupture of membranes and washing hands before handling the baby

## Discussion

### Incidence of neonatal sepsis

Our study provides community based evidence of high incidence of clinical neonatal sepsis. This high incidence in an urban community which is relatively well served with health facilities and with good health care seeking indicators is very disturbing. It is noteworthy that the rate of health facility delivery and delivery under skilled supervision in our study was very high (93.8%) compared to the national rate in Uganda reported in the Uganda Demographic Health Survey (UDHS) of 2011 (57%) and the rate of Antenatal Care (ANC) attendance was likewise better [8]. It would therefore have been expected that the rates of neonatal sepsis in this study be much lower. A community study conducted in India reported similarly high incidence of clinical neonatal sepsis of 17% [14]. However in that study the rate of health facility delivery was very low at only 5%, and thus the possible reason for the high incidence of clinical neonatal sepsis. The finding in our study therefore questions the quality of antenatal services offered with regards to teachings on newborn care practices and other ways of preventing acquisition of sepsis and the techniques of infection prevention in the health facilities during delivery. The fact that more neonates developed sepsis within the first 7 days of life further underlines this problem. This challenge is worsened by the inadequate follow up of neonates since there is delayed postnatal review and not much is known about the state of the neonate from the time of birth till the next immunisation schedule at 6 weeks of life.

The results from our study could still be an underestimation of the incidence of neonatal sepsis since the study was not designed to ascertain sepsis in neonates who died prior to enrolment. In addition the follow up and the health education provided by the study team could have resulted in lower rates of neonatal sepsis.

### Low neonatal mortality in the study

Although the incidence of sepsis was high the neonatal mortality in this cohort was astoundingly low at 0.003% compared to the national NMR in Uganda of 27 per 1000 live births and the global neonatal mortality of 29 per 1000 live births [1, 2]. Given the fact that most of the neonates enrolled were over 7 days of age this study could have missed the infants who died in the early neonatal period, which is the most risky period. Thus this neonatal mortality is low because of the low risk cohort. Although this is the most likely reason for the low mortality, it is important to note that this study closely followed up the neonates, providing early referral in case of clinical features of sepsis. This may suggest that with low cost interventions involving close follow up of neonates in the community, either through domiciliary care or VHT system, effective health education on identification of danger signs and early referral to the health facilities it may be possible to reduce the NMR in low income countries.

### Factors associated with neonatal sepsis

Neonates born to mothers who had PROM more than 18 h prior to delivery were 13 times more likely to develop sepsis than neonates of mothers who had rupture of membranes at birth or less than 18 h prior to delivery. PROM is a risk factor of ascending infection to maternal uterine cavity with resultant infection of the foetus [16]. Studies have elucidated the association between neonatal sepsis and PROM. Jayan et al. (2008) found that neonates born to mothers with PROM were 15 times more likely to develop neonatal sepsis than neonates of mothers who had rupture of membranes at birth or less than 18 h prior to delivery [10]. Although prophylactic antibiotics are given to these risk groups, many mothers do not receive it because of delayed health seeking on noticing leakage of fluid.

Neonates born to mothers who did not receive any paternal financial support were 4 times more likely to develop sepsis compared to those whose mothers received financial support from the fathers. In Uganda health insurance is limited to only a small proportion of the middle and upper social class. The majority of the population seek health care from public health facilities which are usually resource constrained or private facilities which charge varying rates of fees. Most mothers are full time housewives with no source of income; thus they depend on their spouses for financial support for transport, medical related bills and general upkeep of the family. Lack of financial support may affect the mother’s choice of place of delivery and her entire welfare during antenatal, delivery and postnatal. Lack of financial support would lead to poverty and the interplay between poverty and disease has been described [17]. This could explain the reason for the higher rates of sepsis among neonates whose mothers did not receive financial support from the fathers. Studies in Uganda by Waiswa et al. and Byaruhanga et al. have revealed the role of paternal support in determining place of delivery and other aspects of care [18, 19]. The MoH of Uganda emphasizes paternal participation in the care of the mother and newborn during pregnancy and at delivery. This finding further underscores the need to encourage and support women in low income countries to be in position to take care of their health needs.

Neonates born to mothers who washed their hands with soap before handling their baby were less likely to develop sepsis compared to those whose mothers did not wash their hands before handling the baby at bivariate analysis. This study was conducted in a densely populated urban community with areas characterised by uncontrolled developments and slum conditions. These communities generally have limited access to safe clean water which may affect the habit of hand washing. In addition, some cultural beliefs discourage washing hands by visitors before carrying the babies as it is believed that the blessings are washed away. The association between neonatal sepsis and maternal hand washing was reported in an Indian study which showed that maternal hand washing was associated with lower neonatal mortality. Neonates who were exposed to both birth attendant and maternal hand washing had 41% lower mortality [9]. These results emphasise the need for continued health education on the importance of hand washing and advocacy to the government to improve access to safe clean water to all communities.

Although appropriate antenatal care and clean delivery in health facilities have been shown to be associated with reduced sepsis our study did not show this relationship [20, 21]. This was possibly due to the high number of health facility delivery in our study with a small comparison group. In addition our study did not show association between maternal level of education and neonatal sepsis as has been revealed by other studies [22].

### Study limitations

The study was not designed to ascertain the cause of death in neonates who died prior to enrolment. Infants with early neonatal sepsis who were born in health facilities or those born at home and admitted in health facilities were missed, which could have affected the incidence reported. Most of the neonates enrolled were over 7 days of life, thus this was a low risk cohort. In addition the patients who were lost to follow up could have affected the results since their outcome was not ascertained. The above factors may have caused an underestimation of the incidence of neonatal sepsis and the mortality in this cohort..

A diagnosis of clinical neonatal sepsis was made using the IMNCI criteria which has a high sensitivity and low specificity. This could cause over-diagnosis of neonatal sepsis.

Although probability proportional to size was used to sample the zones in study area, the mother-infant pairs were recruited consecutively which could have caused bias.

## Conclusion

The population based incidence of neonatal sepsis in this urban community was disturbingly high despite the relatively good accessibility to health facilities and much better health care seeking indicators than the national picture. This indicates that the quality of antenatal, perinatal and postnatal care offered in the health facilities with regards to infection prevention is sub-optimal. The current mode of promotion of the essential newborn care practices by the WHO and the Ministry of Health of Uganda have not translated into improved uptake of the practices; and yet the study has shown that simple practices like hand washing would lead to reduction in neonatal sepsis. The presented cohort has to be defined as "low risk", partially explaining the low mortality rate. Additionally the close follow up of the neonates induced by the study protocol provided early referral incase of clinical features of sepsis improving the chance of survival. However it may also suggest that the neonatal mortality in developing countries may be reduced with promotion of simple low cost interventions like close community follow up of neonates using village health teams or domiciliary care; health education on identification of danger signs and early referral. Paternal involvement in the care of the mother and infant should be emphasized and women should be supported to take charge of their health.

## Abbreviations

IMNCI: Integrated Management of Newborn and Childhood Illnesses
MoH: Ministry of Health
NMR: Neonatal mortality rate
PROM: Prolonged rupture of membranes
SSA: Sub-Saharan Africa
UDHS: Uganda Demographic Health Survey
VHT: Village health teams
WHO: World Health Organisation
